# Optofluidic flow meter for sub-nanoliter per minute flow measurements

**DOI:** 10.1117/1.JBO.27.1.017001

**Published:** 2022-01-31

**Authors:** Jalal Sadeghi, Paul N. Patrone, Anthony J. Kearsley, Gregory A. Cooksey

**Affiliations:** a University of Maryland, Department of Chemistry and Biochemistry, College Park, Maryland, United States; b National Institute of Standards and Technology, Microsystems and Nanotechnology Division, Gaithersburg, Maryland, United States; c National Institute of Standards and Technology, Applied and Computational Mathematics Division, Gaithersburg, Maryland, United States

**Keywords:** sub-nanoliter per minute flow, optofluidics, flow meter, calibration, photobleaching, molecular diffusion

## Abstract

**Significance:**

Performance improvements in microfluidic systems depend on accurate measurement and fluid control on the micro- and nanoscales. New applications are continuously leading to lower volumetric flow rates.

**Aim:**

We focus on improving an optofluidic system for measuring and calibrating microflows to the sub-nanoliter per minute range.

**Approach:**

Measurements rely on an optofluidic system that delivers excitation light and records fluorescence in a precise interrogation region of a microfluidic channel. Exploiting a scaling relationship between the flow rate and fluorescence emission after photobleaching, the system enables real-time determination of flow rates.

**Results:**

Here, we demonstrate improved calibration of a flow controller to 1% uncertainty. Further, the resolution of the optofluidic flow meter improved to less than 1  nL/min with 5% uncertainty using a molecule with a 14-fold smaller diffusion coefficient than our previous report.

**Conclusions:**

We demonstrate new capabilities in sub-nanoliter per minute flow control and measurement that are generalizable to cutting-edge light-material interaction and molecular diffusion for chemical and biomedical industries.

## 1 Introduction

Many new miniaturized systems in drug delivery handle sample flows in the range of micro- to nanoliters per minute. For example, several critical medicines are administered from an implantable continuous infusion pump at starting doses of 70  nL/min.^1^ In organic chemistry and catalysis, some microreactors operate with flow rates down to 25  nL/min.^2^ Advanced analytical instrumentation, such as high-performance liquid chromatography methods, go even further, using effluent flow rates as low as 5  nL/min.^3^^,^^4^ Furthermore, micro- and nanodispensing (e.g., microdroplets) are utilized increasingly to compartmentalize and process small amounts of liquid for a wide range of applications, including analysis of single bacteria and compartmentalization of biomolecular reactions.^5^^–^^8^

These critical applications have led to an increased need for more accurate measurement of low flows. Microliter per minute flow rates have traditionally been measured by thermal methods, gravimetry, or front tracking. Many flow measurement techniques are not traceable in-and-of-themselves and thus must be calibrated by another method. Calibration usually involves a gravimetric flow measurement (e.g., rate of mass accumulation versus time), which itself has limited accuracy in the nanoliter per minute regime. Low-flow measurements break down when mass accumulation rates are on the same order as errors due to evaporation or external forces on the microbalance, such as from capillary forces against tubing.^9^^,^^10^ In addition, as bulk fluid flow becomes smaller, competition with factors such as diffusion often blurs the resolution of ever-smaller flow displacements.^11^ These factors add up to significant increases in relative measurement uncertainties below 1  μL/min. For example, a commercially available thermal flow meter has uncertainties at different flow rates of 2% at 1.25  μL/min, 4% at 620  nL/min, 8% at 250  nL/min, and above 15% below 100  nL/min.

Integrated optical elements in microfluidic (optofluidic) designs have added unique sensing potential for a variety of label-free and labeled technologies, such as optomechanical transducers, interferometers, fluorescence measurements, and so on.^12^^,^^13^ Nanochannels, nanotubes, or nanopore structures have been employed to enhance advection over diffusion, although they have limited dynamic range and undetermined accuracy. Nanostructured channels have other special considerations, such as vastly increased viscosity near the channel walls.^14^

We have previously reported on an optofluidic flow meter capable of traceable flow measurements down to 10  nL/min using a similarity solution and associated scaling. The method utilizes photophysics of photobleaching to calculate the flow rate from a relationship between fluorescence efficiency and dosage of light sustained by fluorophores as they pass through an interrogation region.^15^ By itself, this efficiency-dosage relationship only allows flow measurement on a relative scale and must be calibrated using a reliable absolute method. Previously, we transferred calibration to our system from a thermal flow meter calibrated to 5% relative uncertainty at 1.1  μL/min. Here, we construct a direct relationship from the flow meter to a traceable gravity-based flow system in which flow rates change linearly with the height of the fluid reservoir. This paper demonstrates nearly 10-fold improvement over previous work in absolute accuracy (to below 1  nL/min) using fluorescein-functionalized molecules with smaller diffusion coefficients. In addition, by directly linking measurements to a microbalance, we improved calibration of the flow controller used to test the system and to determine its fluidic resistance (${\text{conductance}}^{-1}$).

## 2 Principles of Measurement of Optofluidic Flow Meter

Optofluidic flow meter measurements are based on a set of general assumptions: (1) fluorophore advection >> fluorophore diffusion; (2) fluorescence increases with the intensity of excitation light; (3) on average, fluorophores bleach after being exposed to a given number of photons; (4) a flow measurement can be performed absent any detailed information about the physics of fluorescence, the flow profile, or the system geometry^16^; and (5) the bleaching rate depends on the intensity of excitation light. We empirically determined that a nonlinear correction of 1.26 was necessary to correct excitation power such that fluorescence emissions matched for all conditions having equivalent dosages.^15^

We previously developed a model wherein these assumptions were used to define a scaling relationship that quantifies the flow rate $v_{v}$ in terms of the dosage ξ of light received.^15^^–^^17^ The key idea of this approach relies on fluorescence efficiency $I_{f}$, i.e., the fluorescence power measured per unit laser power, being a one-to-one function of the dosage, i.e., $I_{f}=I_{f}(\xi )$. As ξ depends directly on the laser power and is inversely proportional to the flow rate, there is a universal reference curve such that, for any flow rate, measurements of $I_{f}(\xi )$ can be mapped onto this reference by appropriately scaling the laser power.^12^ Notably, this process enables determination of $v_{v}$ from a measurement if the laser power and scaling factor are known. Accurate estimation of an unknown $v_{v}$ hinges on determining the universal form of $I_{f}(\xi )$ via calibration using a known flow rate. Importantly, this measurement process, while accurate, must work within constraints imposed by the system components. For example, the error in the model—and thus in the data analysis—is approximately inversely proportional to an effective Peclet number $P_{\mathrm{eff}}=v_{v}/LD$, where L is the characteristic length of the interrogation region and D is the diffusion coefficient of the fluorescent molecules [see assumption (1) above]. Of note, the error becomes large as $v_{v}$ becomes small. Decreasing the diffusion rate D associated with the fluorophores therefore extends the range of flow rates that can be accurately measured; we consider such modifications in this work using larger (and thus more slowly diffusing) fluorescent molecules. In addition to $P_{\mathrm{eff}}$, the uncertainty in the flow rate calibration used to construct the reference curve has the potential to control the relative error of our flow meter at all flow rates.^12^

This work focuses on controlling and quantifying the uncertainties of the calibration system that underlie the reference curve used to determine unknown flow rates. We use a gravimetric flow controller to create known flow rates. In particular, we employ the Hagen–Poiseuille equation to determine the volumetric flow rate ($v_{v}$) from the linear relationship of $dH=(H-H_{0})=v_{v}R/g_{n}\rho$. Here, dH is the height relative to a reference height, $H_{0}$ is the height where $v_{v}=0$, R is the resistance of the microfluidic channel to flow, and $g_{n}$ is the standard acceleration due to gravity. At the outset, the device resistance is unknown and must be determined from a primary standard. For this purpose, we implemented a gravimetric flow meter. Section 3 illustrates how uncertainty in this gravimetric flow meter propagates through our flow controller into reference flow rates used to calibrate the microfluidic system. Several observations facilitate the technical discussion that follows. For the flow controller, both $H_{0}$ and R are unknown and determined simultaneously. This can be accomplished by a two-point calibration of the flow rate at two different heights. It is desirable to maximize height differences such that the smallest possible uncertainty in the primary gravimetric measurement gets passed through to the flow controller in determining R. First, $H_{0}$ is found to be the height of the liquid reservoir that leads to a critical value in fluorescence as the flow transitions from positive to negative.^7^ Next, the height of the reservoir is set to the maximum height (1 m), which sets the maximum flow rate through the device. Measurement of the mass flow onto the gravimetric system then commences. In this analysis, uncertainty of the height measurements is excluded, as the positional error associated with the vertical stage is expected to be on the order of 2  μm, or ≪1% of the range of flows generated by the flow controller. The uncertainty of the $H_{0}$ is discussed below.

According to above assumptions (4) for this analysis, defects in channel geometry or surface roughness should not affect the measurement or scaling model. As derived previously,^16^ the model requires minimal knowledge of system geometry or any other information about the physics of fluorescence or flow profile. Thus, even if surface features were to cause streamlines to be locally bent, low Reynold’s numbers assure that streamline positions (and model) are stable.

### 2.1 Materials, Microchip Fabrication, and Measurement Procedure

This optofluidic device was fabricated as reported.^15^ Briefly, templates for devices containing both microfluidic and waveguide channels were created using SU8 (SU-8 2075, Kayuka Advanced Materials) on silicon wafers. (Identification of commercial products does not imply recommendation or endorsement by the National Institute of Standards and Technology (NIST). The materials and equipment used may not necessarily be best for the purpose.) The templates were directly written using a maskless aligner at the Center for Nanoscale Science and Technology at NIST. Devices were cast in poly(dimethylsiloxane) (PDMS) (Sylgard 184, 10:1, Dow Corning) by pouring PDMS over silanized templates and curing for 3 h at 70°C. The system design included the main flow channel, ports, debris traps, and channels that serve as waveguides. Two emission waveguides were positioned upstream and downstream of the excitation waveguide at oblique angles of ≈29  deg. Additional channels were designed around the waveguide channels and filled with opaque PDMS (Sylgard 170, 1:1, Dow Corning) and cured for 1 h at 70°C to block stray scattered or reflected light. Next, the channels for excitation, emission, and transmission waveguides were filled with optical adhesive (refractive index of 1.56). Cleaved multimode optical fibers (105  μm core and 125  μm cladding diameters) with 0.22 numerical aperture were inserted into the waveguides prior to curing with UV light for 2 h.

**Fig. 1:**
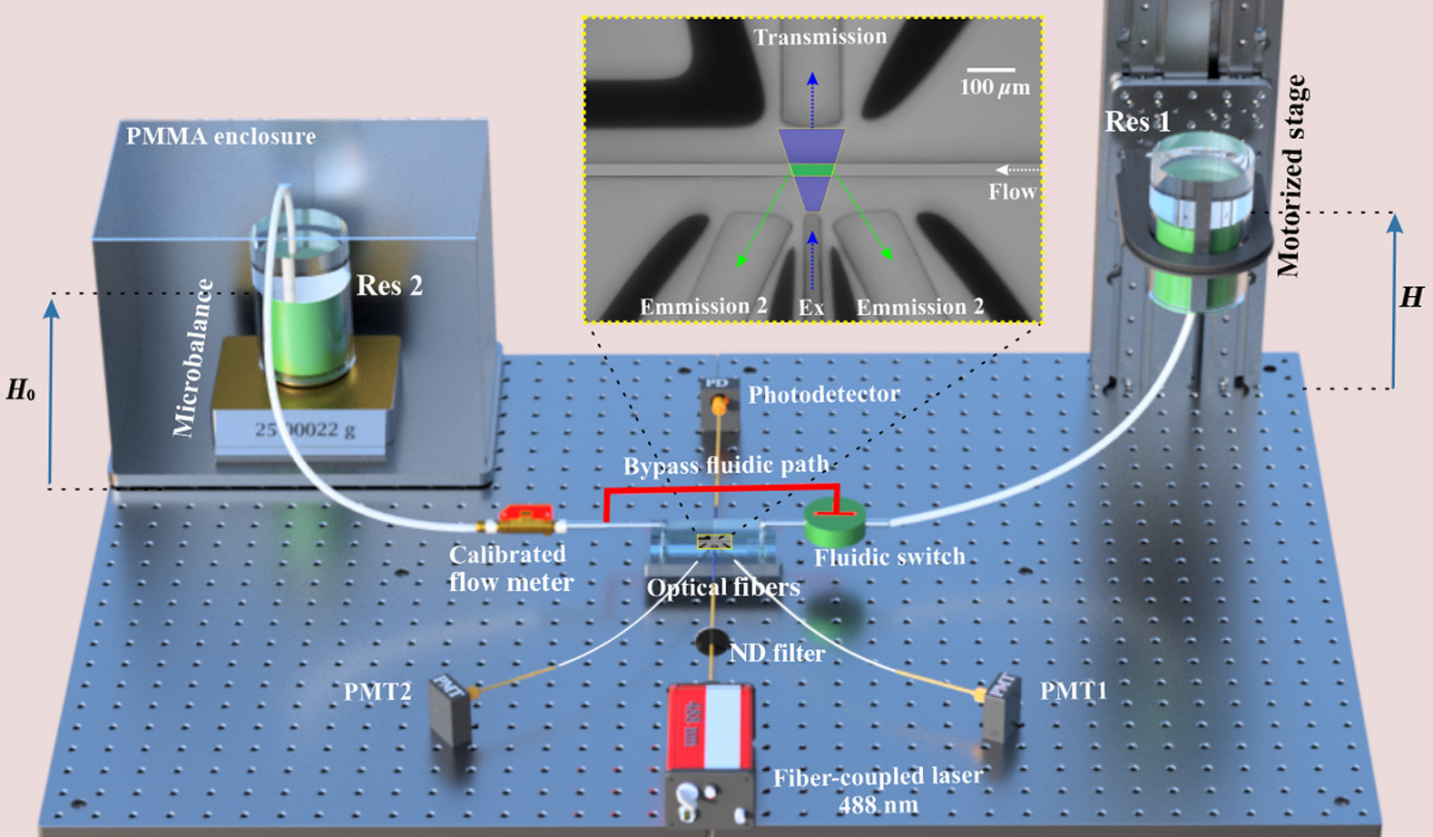
Schematic showing the fluidic and optical elements of the optofluidic flow meter system. The height difference ($H-H_{0}$) of the source reservoir (Res1) on the motorized stage and the collection reservoir (Res2) on the microbalance determine the pressure driving the flow. The inset shows an image of the optical interrogation region, including the microfluidic channel carrying the flow (right to left), excitation light (blue, 488 nm), and waveguides that collect emitted light (green, ≈ 520  nm) and transmitted light.

A schematic sketch of the optofluidic flow meter setup, including fluidic and optical connections and the gravimetric calibration system, is shown in Fig. 1. Fluid flow was controlled by adjusting the height difference between a source and collection reservoir using a long-travel high-precision vertical stage (customized LS-270, Physik Instrumente). The stage enabled control of hydrodynamic pressure of the water column over 1-m distance with 2-μm absolute accuracy. Mass flow was tracked by time-dependent recording of the mass accumulation in a reservoir on a microbalance. The reservoir was covered with oil to reduce evaporation. We inserted three-way fluidic switches on each side of the microchip to enable a rapid short circuit for setting the position of zero flow and validating the critical point in the signal for zero flow.^7^ Flow rates were also tracked in real time with one of two commercial thermal flow meters.

Laser light was coupled via a multimode optical fiber into a neutral density (ND) filter cube and then into the excitation waveguide in microfluidic devices. A ND filter with nominal 10% transmittance was added to the excitation path to extend the power range of the optofluidic system to lower levels (Fig. 1). The excitation waveguide was connected to a fiber-coupled diode laser (LuxX 488 nm, 200 mW, Omicron-Laserage), and the emission and transmission waveguides were connected to photomultiplier tubes (PMTs) (H10721-20, Hamamatsu) and silicon photodetectors (918D-SL-OD2R, Newport Corp.), respectively. Emission detectors were further fitted with bandpass filters for fluorescein emission (520  nm±40  nm). Transmitted light was collected to normalize the fluorescence collection to incident laser power. We call this ratio fluorescence efficiency. Flow measurements were conducted using either 25  μmol/L fluorescein sodium salt (30181, Sigma-Aldrich) or 12.5  μmol/L FITC-dextran with 70 kDa mean molecular mass (FD70S, Sigma-Aldrich) dissolved in a borate buffer (pH 8.5).^18^ After calibrating the flow rate to the height through fluidic resistance, the scaling error associated with the fluorescence efficiency versus dosage relationship was compared by scanning measurements across a series of flow rates and laser powers.^15^

## 3 Calibration and Uncertainty Analysis

As discussed in Sec. 2, the accuracy of low-flow measurements depended on the uncertainty of calibration and the method of flow generation. We used a gravimetric system for calibration, the uncertainty analysis of which was derived from a similar system developed for microflow measurements.^9^ Briefly, the measurement principle of dynamic gravimetric methods for the standard is based on transferring the mass into a reservoir on the microbalance (Res2) during the measurement time interval. The flow equations and guiding uncertainties of a gravimetric measurement system are described in more detail in fundamental studies by Melvad (2010),^19^ Bissig (2015),^20^ and Wright and Schmidt (2015, 2019).^9^^,^^21^ Similarly, we describe the governing equations for volumetric flow and measurement uncertainty for our design as

$$v_{v}+\varepsilon_{v}=\frac{(H-H_{0})g_{n}\rho_{\mathrm{Liq}}}{R+\varepsilon_{R}}=(\frac{1}{\rho_{\mathrm{Liq}}})\frac{dm_{\mathrm{Liq}}(t)}{dt},$$ (1a)

$$\frac{dm_{\mathrm{Liq}}(t)}{dt}=[\frac{dm_{\mathrm{Meas}}(t)}{dt}[\frac{1-\frac{\rho_{\text{Air}*}}{\rho_{\mathrm{Std}}}}{1-\frac{\rho_{\text{Air}}}{\rho_{\mathrm{Liq}}}}]-\frac{dm_{\text{Drift}}(t)}{dt}+\frac{dm_{\mathrm{Evap}}(t)}{dt}+\frac{dm_{\mathrm{ST}}(t)}{dt}+\varepsilon_{\overset{˙}{m}}],$$ (1b)

where ($dm_{\mathrm{Liq}}/dt$) is the liquid mass flow through the system from Res1 into Res2, $\varepsilon_{v}$ is the the flow rate uncertainty, and $\varepsilon_{R}$ is the uncertainty in the device resistance. All quantities of interest are ultimately determined from the measured mass flow rate, $dm_{\mathrm{Meas}}(t)/dt$, which is corrected for buoyancy based on the terms $\rho_{\text{Air}*}$, $\rho_{\text{Air}}$, $\rho_{\mathrm{Liq}}$, and $\rho_{\mathrm{Std}}$, the density of air assumed by the balance ($0.0012\;g/{\mathrm{cm}}^{3}$), the density of air at the time of measurement, the density of water, and the density of the calibration mass, respectively.^9^ The mass flow rate is further corrected by factors for microbalance drift, $dm_{\text{Drift}}(t)/d$t; uncertainty due to evaporation, $dm_{\mathrm{Evap}}(t)/dt$; uncertainty due to surface tension effects, $dm_{\mathrm{ST}}(t)/dt$; and a factor, $\varepsilon_{\dot{m}}$, which accounts for all sources of uncertainty associated with the aforementioned physical processes. Our primary goals in this section are to (1) identify and quantify the various contributions to $\varepsilon_{\dot{m}}$ and (2) demonstrate how $\varepsilon_{\dot{m}}$ affects uncertainty in the flow rates used to calibrate the optofluidic flow meter.

We address goal (2) first. Examining Eqs. (1a) and (1b), we note that all terms except $\varepsilon_{\dot{m}}$, $\varepsilon_{R}$, and $\varepsilon_{v}$ represent deterministic quantities, i.e., those associated with measurement values, whereas the uncertainties can be interpreted as random variables. Thus, in these equalities it is meaningful to consider the deterministic and random parts separately. This leads to the equality $\varepsilon_{v}=\frac{\varepsilon_{\overset{˙}{m}}}{\rho_{\mathrm{Liq}}}$. Conveniently, $\varepsilon_{R}$ does not appear in this equation because it is inversely proportional to both uncertainty terms. Our task amounts to estimating $\varepsilon_{\dot{m}}$.

In the next sections, we describe estimating individual contributions of the physical processes to $\varepsilon_{\dot{m}}$, which include four main components: (1) the mass balance repeatability (ε(Δm)) and drift ($\varepsilon (m_{\mathrm{Bal}})$), (2) buoyancy correction, (3) uncertainty due to evaporation ($\varepsilon (m_{\mathrm{Evap}})$), and (4) surface tension effects ($\varepsilon (m_{\mathrm{ST}})$).

### 3.1 Mass Balance Repeatability and Drift

We first consider the uncertainty due to resolution and repeatability of a fixed mass, which we calculate from specified resolution of 10  μg and repeatability of 15  μg.^9^ Following Schmidt and Wright’s derivation, for the difference of two mass measurements separated by some time interval, the value is $\varepsilon (\Delta \overset{˙}{m})=(\surd 2{\left(10^{2}+15^{2}\right)}^{1/2})/10\;\min=2.5\;\mu g/\min$, where the sampling interval is 10 min.

**Fig. 2:**
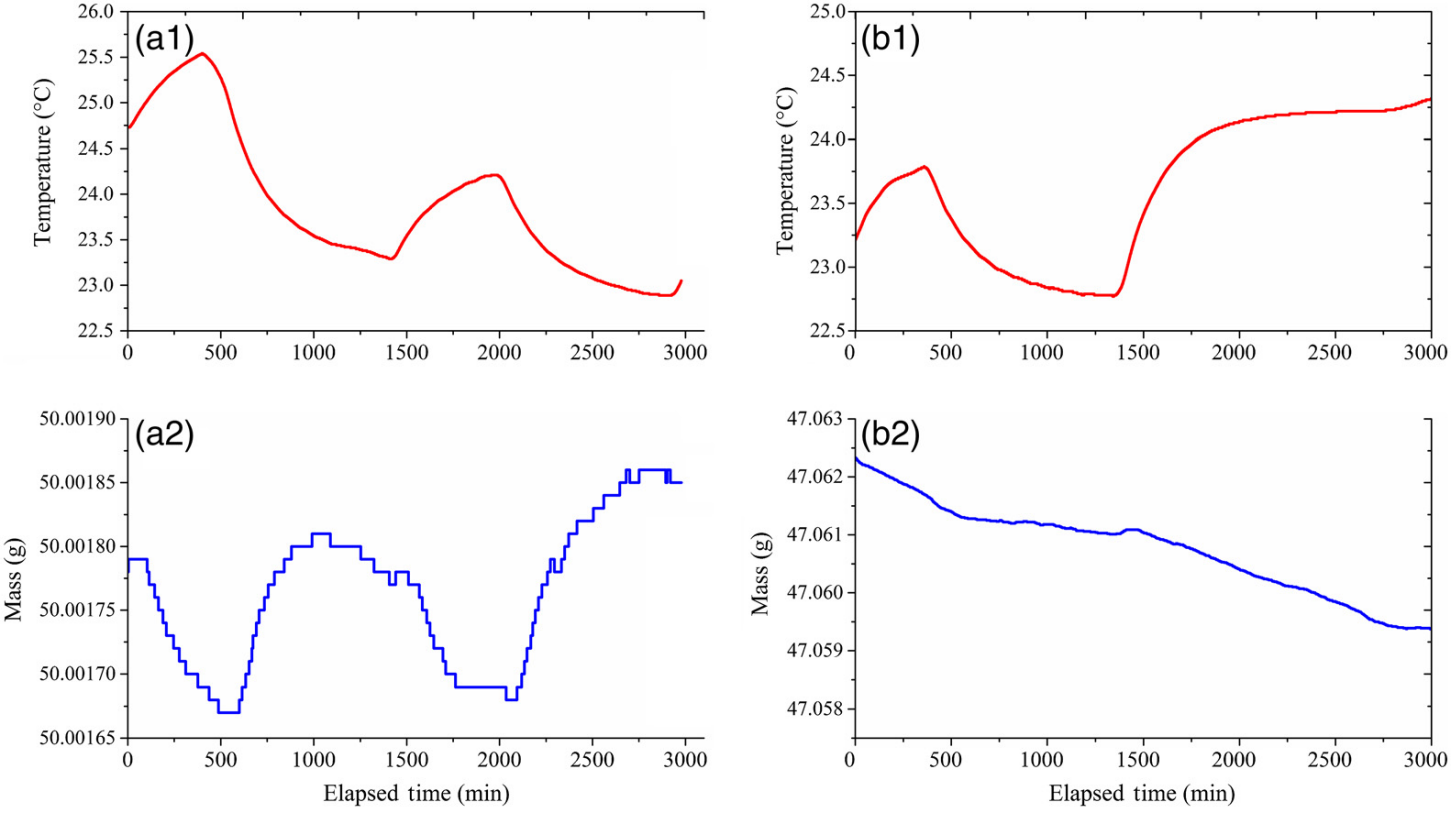
(a1) and (a2) Temperature and microbalance readings with a 50-g metric weight over nearly 50 h. (b1 and b2) Temperature and microbalance readings of Res2 at zero-flow conditions versus time.

Next, we evaluate the stability of 50 g tare weight over several days, while also recording room temperature (5240 TECSource; Arroyo Instrument), as shown in Fig. 2. To reduce static electricity and air flow modifications to the measurement stability, the microbalance was placed in a polymethyl methacrylate enclosure, which resulted in a maximum temperature variation of ±0.15°C/h. A typical experiment lasts <5  h, so a conservative estimate of the balance drift is expected to be bounded by the maximum mass derivative over any 10 min period, which we found to be 7  μg. This corresponds to 0.7  μg/min or a maximum error of roughly 0.7  nL/min. Looking more closely, we see that mass fluctuations are inversely related to temperature fluctuations, so an improvement in drift estimate is available by accounting for temperature variations. However, a conservative combination of these two contributions to the flow uncertainty gives:

$$e({\overset{˙}{m}}_{\mathrm{Bal}})=[{\left(e(\Delta m)/\Delta t\right)}^{2}+{\left(dm_{\text{Drift}}/dt\right)}^{2}{]}^{0.5}=[{\left(2.5\;\mu g/\min\right)}^{2}+{\left(0.7\mu g/\min\right)}^{2}{]}^{0.5}\approx 2.6\;\mu g/\min$$

### 3.2 Evaporation Uncertainty

Evaporative loss is a significant component in uncertainty calculation of small flows,^9^ and evaporation from the collection vial affects the uncertainty of the gravimetric calibration. In our case, evaporation effect has been significantly reduced by covering the liquid in both reservoirs with a low-volatility oil. In Fig. 2(b2), we show that the maximum rate of evaporation under laboratory conditions is likely to be roughly 2  μg/min (equivalent to effective error of 2  nL/min of water loss). Evaporation is also expected to increase uncertainty to our flow controller as it leads to height changes. Symmetry of reservoirs on each side of our device, however, results in nullification of uncertainties due to effects such as evaporation. Thus, evaporation is only considered in the calibration of the flow controller (at maximum height) and is not expected to add additional uncertainties to the flow controller while in operation.

### 3.3 Uncertainty due to Surface Tension Effects

Surface tension on the needle entering the collection vial can induce error in the gravimetric calibration as stick-slip phenomena lead to intermittent variation in the forces on the microbalance as the liquid rises in the reservoir due to flow. Previously, Schmidt and Wright^9^ estimated a maximum uncertainty of 65  μg for this effect (given a similarly sized needle D≈1.8  mm). We have not studied the extent of the contact angle change nor the frequency of stick-slip phenomena, although the latter is expected to be slow given the large surface area of the collection vial. However, given that we do not observe random variations of a degree larger than evaporation over a 5-day observation period (at a maximum rate of 2  nL/min), we propose that, under the conditions of this test, the time-average errors due to stick-slip phenomena are on the order of evaporation or smaller. Further improvements in our uncertainty analysis warrant more careful study of this phenomenon.

### 3.4 Propagation of Uncertainty into the Flow Controller

The combined uncertainty of the gravimetric calibration of flow is

$$\varepsilon_{\overset{˙}{m}}\approx {\left[{\left(2.6\right)}^{2}+(2.0{)}^{2}+(2.0{)}^{2}\right]}^{0.5}\approx 3.8\;\mu g/\min,$$

which gives $\varepsilon_{v}\approx 3.8\;\mathrm{nL}/\min$ (k=1), as described above, with the assertion that $\rho_{\mathrm{Liq}}$ does not vary by more than 1% under our experimental conditions. We use this uncertainty to calibrate the height of the flow controller through R in the Hagen–Poiseuille equation.

### 3.5 Additional Uncertainty Components of the Optofluidic Measurement System

Once calibrated at a high flow rate, e.g., 1000  nL/min (≈0.8% relative uncertainty, k=2), the measurement process of the optofluidic flowmeter has a total uncertainty, $\varepsilon_{\mathrm{FM}}$, which includes additional sources of uncertainty. The scope of this paper is not meant to fully explore the uncertainty components of $\varepsilon_{\mathrm{FM}}$ but rather to estimate its maximum extent. Briefly, however, we describe some key components.

Pressure controls the flow rate through the system proportional to R; thus uncertainty in system pressure, $\varepsilon_{P}$, proportionally affects uncertainty in the flow measurement. Drift in the height calibration as flow moves liquid from one reservoir to another contributes to $\varepsilon_{P}$, and this can be modeled. However, the height change is very small over most measurement intervals. For example, given a 30 mm diameter reservoir, flow at 1  μL/min induces a height change of ≈1.5  μm/min, which when scaled by R, leads to a maximum accumulating error of ≈4  (pL/min)/min.

Bubble formation within the microchannel can also contribute to flow uncertainty. Practically, microbubbles increase R by reducing the cross section of the channel (and can be modeled as a proportional pressure drop). Microbubble formation is typically well controlled, but it is obvious in our experiments, so such data can be excluded. Generally, we mitigate risk of bubble formation by degassing liquids under vacuum prior to an experiment. In addition, maintaining reservoirs roughly 15 cm above the microfluidic chip provides sufficient backpressure to prevent bubble formation inside PDMS.^22^ Ultimately, we have observed that, when a microbubble is present, it induces periodic oscillations in flow, an indicator that the system is not performing as expected and should be reset or pressurized until the bubble dissolves.

Uncertainty in $H_{0}$ ($\varepsilon_{\text{zero}H}$) depends on factors that include the resolution of the vertical stage (2  μm), power of the laser, thermal/mechanical stability, and ability to resolve the signal when flow is balanced, which itself depends on observation time and diffusion coefficient of the fluorescence reporter. Additional factors, such as mechanical vibrations, especially in the tubing that connects the meter to the flow controller and collection reservoir, can induce uncertainty in flow by causing liquid oscillations through the flow meter. We discovered that using rigid tubing wrapped in flexible tubing reduces such vibrations. Liquid leaks ($\varepsilon_{\text{leak}}$) also affect the measurement uncertainty from calibration; these leaks can occur from mismatched tubing components or cracks around the needle-to-PDMS junctions. In addition, a nontrivial leakage component might be the loss of liquid into the walls of the flow meter itself. The loss of liquid into PDMS is well known^23^; however, it is expected that the loss of water into the device is balanced on each side of the measurement region. In addition, the overall loss of flow around the flowmeter would likely lead to lower-than-expected flow measurements, which were not evident. Nonetheless, the magnitude of uncertainty from leakage remains a topic of future discussion.

The error associated with projecting the fluorescence measurement from a flow calibration onto a dosage curve ($\varepsilon_{\text{dosage}}$) includes notable sources of uncertainty including laser stability, contributions from the PMT stability and noise, approximation of steady state (photobleaching), properties of the fluorescence reporter, and assumptions of linearity of all optical components to intensity. While this list is not exhaustive, it provides quantitative insight as it facilitates discussions about how to improve the measurement technology.

## 4 Results

In a previous manuscript,^15^ we described an optofluidic system capable of dynamic flow measurements to 10  nL/min with relative uncertainty that was scaled down from a calibrated thermal flow meter at a much higher flow rate (where it was at the lower limit of its 5% uncertainty specification). In this paper, we take a twofold approach to improving the measurements.

First, we improve the uncertainty of the flow controller used to drive flow into the optofluidic flow meter. To achieve this result, we incorporate a gravimetric calibration system directly in series with our flow meter bypassing the less accurate but more dynamic thermal flow meter. Using Eqs. (1a) and (1b), we determine the uncertainty of a gravimetric flow meter and how its uncertainty propagates to a flow controller that uses precise control of the height of a source water column to drive flow into the optofluidic system. Effectively, the gravimetric calibration enables determination of fluidic resistance, R, such that flow rates of low uncertainty can be delivered and tested in the optofluidic meter. From the analysis in Sec. 3, we derived the flow rate uncertainty of 7.6  nL/min (k=2). The lowest relative uncertainty in the flow controller calibration is thus realized at the maximum flow rate, which is attained at maximum height of the vertical stage (1 m). For example, we measured a flow rate of 2156  nL/min at 1 m and correspondingly set zero flow at 155 mm using fluidic switches (Fig. 1), resulting in an estimate of R to within 0.8% (k=2). More conveniently, we express the relationship between flow and pressure as conductance, or $R^{-1}=2.55\;(\mathrm{nL}/\min)/\mathrm{mm}$, as it directly relates changes in flow to changes in height of the reservoir. We note that differences in fiber-to-waveguide coupling efficiency could lead to small uncertainty in the zero-flow determination, although the contribution is expected to be very small on the scale of the 1-m column height. Further, once the flow meter is calibrated, we do not expect coupling differences, as long as they are constant, to contribute error to the estimation of flow from the dosage relationship.

**Fig. 3:**
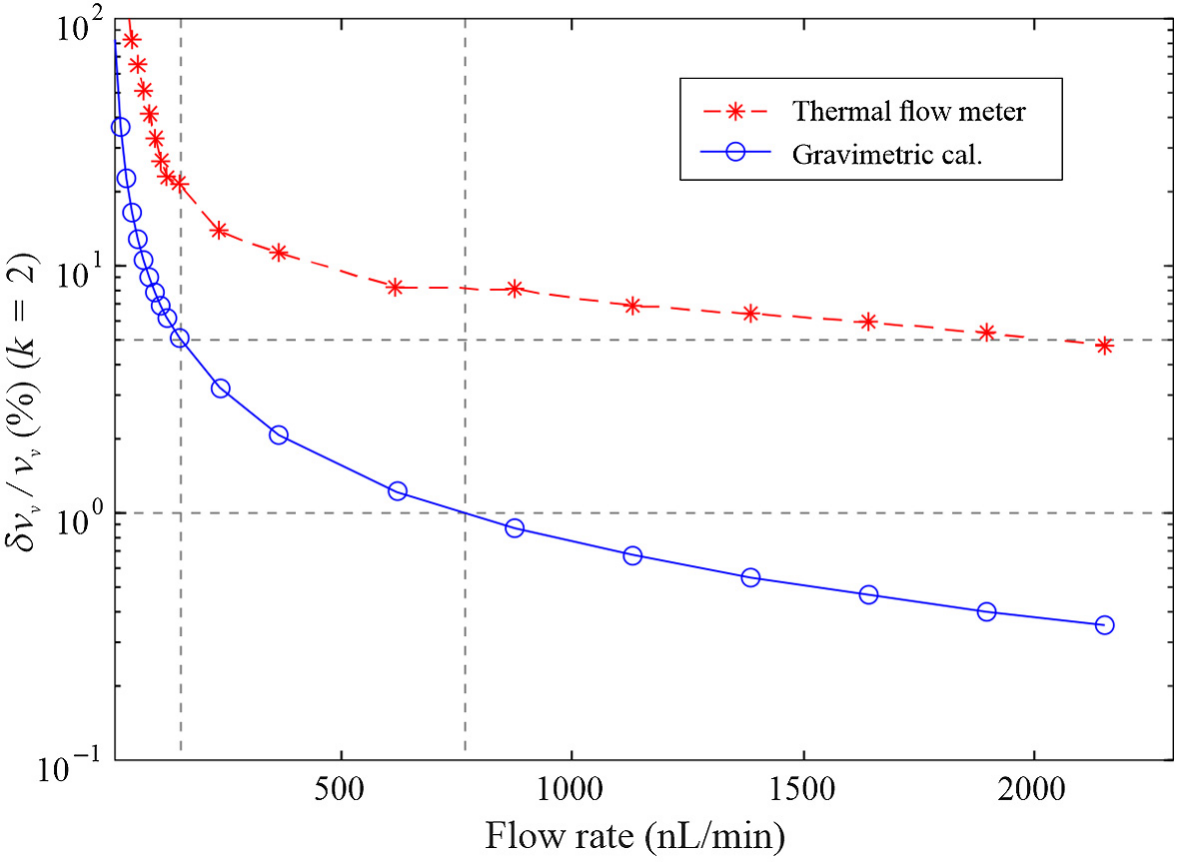
Relative uncertainty (k=2) of volumetric flow using the microbalance to calibrate the flow controller (blue). Comparable uncertainty in flow with thermal flow meter using the calibration (red). Dotted gray lines indicate 1% and 5% relative uncertainty (horizontal) and the corresponding flow rates (vertical) for the gravimetric calibration.

Figure 3 shows the uncertainties (k=2) of the thermal flow relative to the gravimetric calibration. The thermal flow meter showed relative uncertainty of 4.7% at high flow rates and crossed above 10% uncertainty when flow rates dropped below 460  nL/min. By comparison, the gravimetric calibration started at 0.35% uncertainty and grew larger than 5% uncertainty for flow rates below roughly 155  nL/min, which was a 3.9-fold improvement. Even with improvement in the gravimetric calibration applied to the thermal flowmeter, relative uncertainties were 7.6% at 100  nL/min and 81% at 10  nL/min. For comparison, relative uncertainties of the thermal flowmeter from our previous study were >25% below 100  nL/min and >116% below 10  nL/min.

**Fig. 4:**
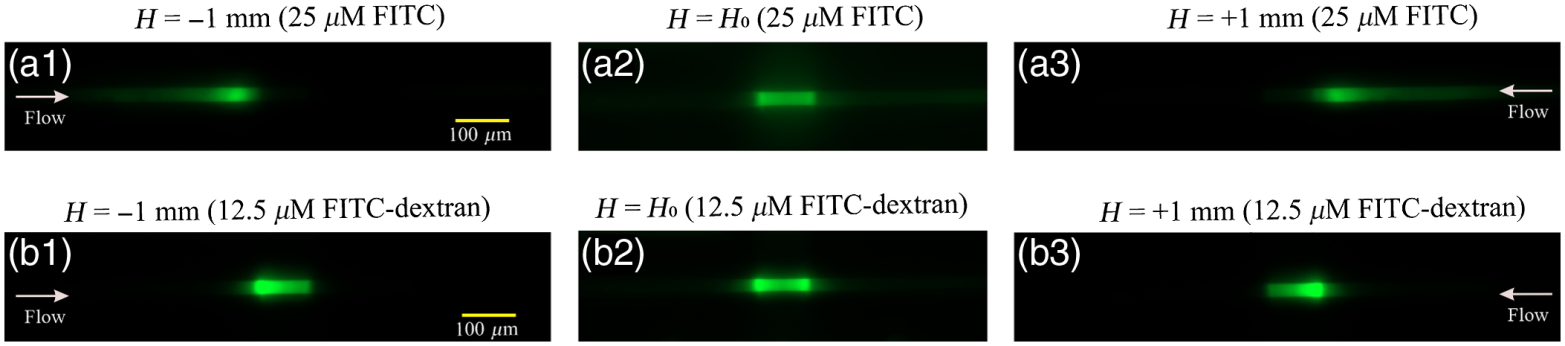
Microscopy images of fluorescence intensity show the difference in the diffusion of fluorescein and FITC-dextran around zero flow after 20 s of bleaching. Initially, the fluorophores present in the channel are bleached by an intense laser beam as schematically shown in the inset of Fig. 1. As shown in figures (a2) and (b2), at zero flow, all fluorescein in the channel is bleached, except for fresh fluorophore diffusing into the edges of the laser path. By increasing the flow to ±2.55  nL/min (±1  mm in height), brighter intensities on the left are used to indicate positive flows, and brighter intensities on the right indicate negative flows.

The second improvement in flow measurements realized by this work was the reduction of the lower limit of dosage rescaling using a higher molecular weight fluorescent molecule. We chose 70 kD molecular mass FITC-dextran, which has a diffusion coefficient approximately 14.3-fold lower than fluorescein alone.^16^ In our previous report,^15^ we used the measurement difference between the upstream and downstream collection waveguides to determine the flow direction and to resolve the point at which the flow was indistinguishable from zero flow. In Fig. 4, we demonstrate the improved clarity separating convection from diffusion with FITC-dextran as the flow controller moves from positive to negative flows. The estimation of zero flow was refined in this report utilizing a new method to rapidly create a zero-flow condition. Rather than relying on resolving the vertical stage position to find the balanced intensity associated with zero flow, we attained “true” zero by introducing a low resistance “short circuit” around the microchip that rapidly balanced the height of the two reservoirs. Thus, equilibrium was achieved in seconds rather than the many minutes required to find photobleaching equilibrium using a height-scanning approach (e.g., Fig. 4).

**Fig. 5:**
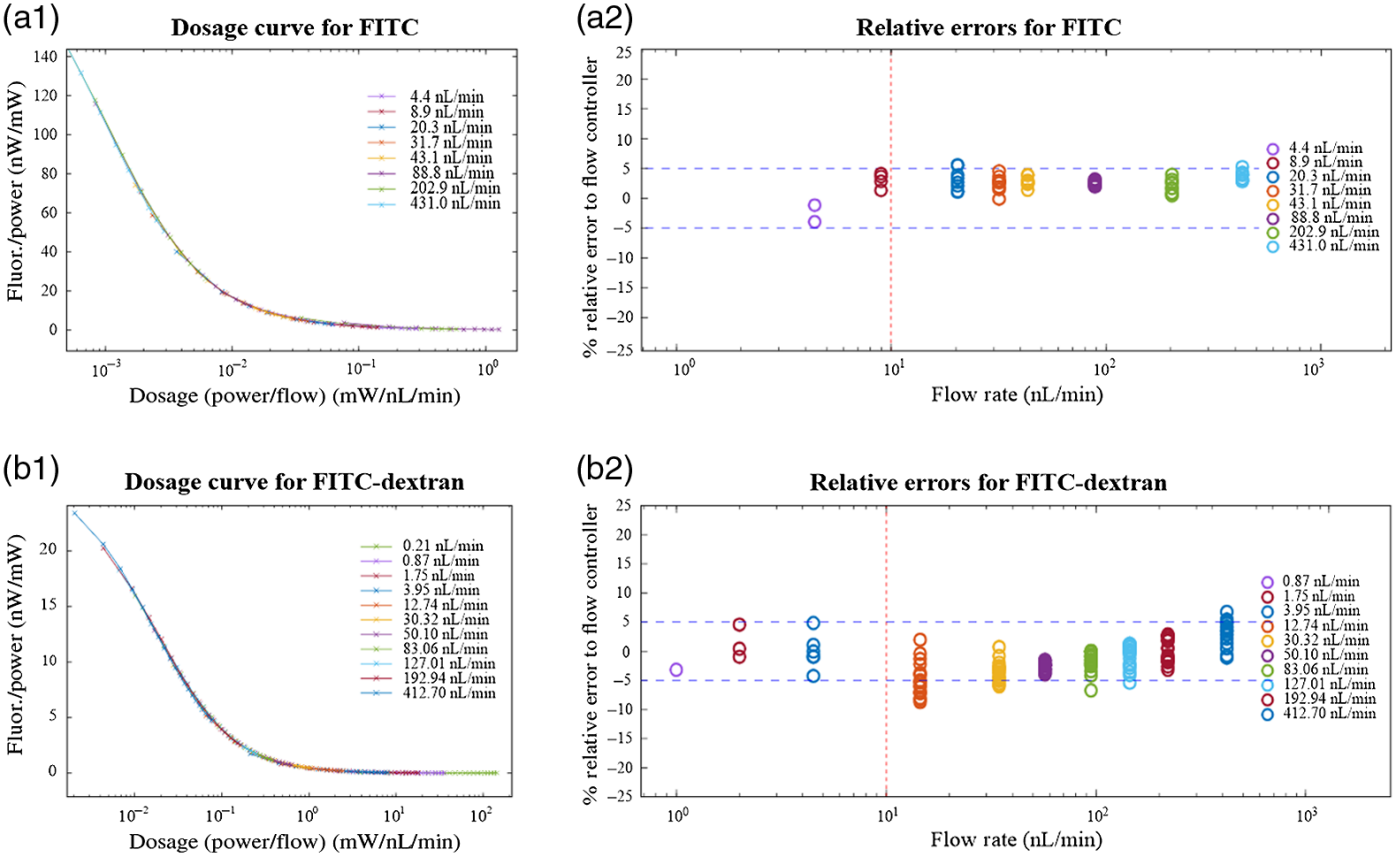
(a1 and b1) Fluorescence efficiency versus dosage plots for laser power and flow rate combinations using fluorescein (top row) and FITC-dextran (bottom row), respectively. (a2 and b2) The relative errors of each condition from the dosage calibration curve are shown where data overlap the curve. Relative errors are the differences in the flow rate between the flow controller and the flow predicted by the calibration curve (for each laser power) divided by the flow rate set by the flow controller.

Validation of the optofluidic flow meter involved collecting fluorescence efficiency over a series of dosage conditions, e.g., combinations of incremental laser powers (from 0% to 100% in 5% or 10% increments) for each of a series of flow rates generated from various heights of the flow controller (e.g., x-axis of Fig. 3). Effectively, this procedure created a series of overlapping dosage-fluorescence efficiency curves (Fig. 5), which were then combined to create a dosage calibration curve. For fluorescein, we chose 20  nL/min as the lower cutoff flow rate for creating the curve. From the dosage calibration curve, we then calculate the error from each dosage condition (Fig. 5). For fluorescein, we found that we could project the calibration curve onto dosages that had overlapping flow rates from the flow controller down to 4.4  nL/min with roughly ±5% maximum relative error. As shown in Fig. 5, for the same flow range, the dosage response curve and relative errors using FITC-dextran projected 5% relative uncertainty down to 0.87  nL/min, which is effectively a 11.5-fold improvement over our previous report and a fivefold improvement compared with the fluorescein data shown here.

## 5 Discussion and Conclusion

In this study, we show significantly improved resolution of our optofluidic flow meter with the same design and same fluorophore as in our previous report, with a few notable changes. The current data were collected with a PMT (rather than a silicon detector), which has more amplification of fluorescent signal in the high-photobleaching range, thus potentially providing greater signal-to-noise values for fluorescence efficiency at low flows. Additionally, we used a buffer with a higher pH, which is known to improve the fluorescence efficiency of fluorescein.^24^

Additional improvements were demonstrated in the resolution of nanoflows using a higher molecular weight fluorescent molecule, as predicted by our previous discussion relating how $P_{\mathrm{eff}}$, which is inversely related to the diffusion coefficient, determines the ability to resolve convection from diffusion.^16^ For fluorescein ($\text{diffusion coefficient}\approx 430\;\mu m^{2}/s$ and $t_{D}=2.9\;s$), $P_{\mathrm{eff}}>20$ down to advection rates of 12.5  nL/min for characteristic dimension of 25  μm. Use of fluorescein-dextran (70 kD, $\text{diffusion coefficient}\approx 30\;\mu m^{2}/s$) permits flowmeter performance at 5% uncertainty to an advective rate of roughly 0.85  nL/min. Though we did not fully realize the improved resolution predicted by the diffusion coefficient, we believe that focusing on factors discussed below, such as photobleaching efficiency, will enable a closer approach to the predicted improvement.

Though we were able to show improvement in uncertainty of the flow controller from 5% to 1%, this did not translate to the dosage rescaling calculation. Effectively, all measurements from 400  nL/min to 1  nL/min that overlap with the dosage calibration curve remain bounded by roughly ±5%. We expect that the uncertainty components described in Sec. 3.5 are thus the likely controllers of total uncertainty. Exploration of the components is beyond the scope of this paper, but we note one factor that requires further study: we observed a change in the effective power-factor correction needed to optimize the dosage rescaling across all conditions. Notably, the power factor required to align the dosage curve shifted from 1.18 to 1.26 in this study, which could be related to a change to borate buffer (pH 8.5) for fluorescein. More detailed investigation of the power factor dependence will be explored in future work.

Future improvements in the measurement system are expected by changing the design and system components to tune the dosage curve for greater sensitivity to photobleaching, as the flow meter does not easily discriminate small changes in fluorescence once the fluorophore is nearly completely bleached (e.g., the dosage curve is flat). Thus, to move to lower flow rates, we plan to explore design changes that reduce photobleaching. Opportunities to reduce photobleaching include designing flow channels with narrower cross sections (to reduce the dwell time of fluorophores in the interrogation region), changing the size of the interrogation region, and choosing fluorophores that are more robust to photobleaching.
